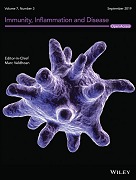# Issue Information

**DOI:** 10.1002/iid3.229

**Published:** 2019-08-08

## Abstract